# Evaluation of the effects of menopause on the quality and quantity of intraoral autogenous block graft donor sites using cone-beam computed tomography

**DOI:** 10.1186/s12903-026-08844-z

**Published:** 2026-06-09

**Authors:** Muhammed Eren Eser, Kami̇l Serkan Ağaçayak, Eli̇f Ağaçayak

**Affiliations:** 1 Department of Oral and Maxillofacial Surgery, Faculty of Dentistry, Yüzüncüyıl University, Van, 65080 Turkey; 2 Department of Oral and Maxillofacial Surgery, Faculty of Dentistry, Dicle University, Diyarbakır, Turkey; 3 Department of Obstetrics and Gynecology, Faculty of Medicine, Dicle University, Diyarbakır, Turkey

**Keywords:** Autogenous block graft, Osteoporosis, Cone-beam computed tomography (CBCT)

## Abstract

**Background:**

Menopause is characterized by the permanent cessation of menstruation and the loss of estrogen-related protective effects on systemic bone metabolism. One of the major consequences of this hormonal alteration is accelerated bone loss and deterioration of bone microarchitecture. Reduced estrogen levels lead to a decrease in bone mineral density and markedly increase the likelihood of osteoporosis development.

**Objective:**

The present study aims to systematically assess postmenopausal changes affecting both the volume of graft material that can be harvested from intraoral autogenous block graft donor regions and the relative radiographic bone density characteristics of these grafts, assessed using CBCT-derived Hounsfield Unit (HU) values.

**Materials and methods:**

The study population comprised patients who had undergone cone-beam computed tomography (CBCT) at the Faculty of Dentistry for various clinical purposes. Participants were categorized into two groups based on menopausal status. The first group included 30 female individuals between the ages of 20 and 40, while the second group consisted of 30 female patients over the age of 55 who had been diagnosed with menopause by a gynecologist, based on prior hormonal assessments (FSH, LH, and estrogen), and who had experienced at least five years since the onset of menstrual irregularity. Information regarding menopausal status, hormonal assessment findings (FSH, LH, and estrogen levels), medical history, and systemic conditions was retrospectively obtained from the patients’ electronic medical records and archived clinical charts. In total, 60 patients were enrolled in the study.

Hounsfield Unit (HU) measurements and graft volume estimations—derived using the Cavalieri principle—were extracted from the CBCT data. In the statistical evaluation, differences between the two independent groups were analyzed using the Student’s t-test for variables with normal distribution, whereas the Mann–Whitney U test was applied to data that did not meet normality assumptions.

**Results:**

The findings indicate that menopausal status, along with its accompanying physiological alterations, adversely affects intraoral autogenous block graft donor regions, specifically the ramus and tuberosity. However, in postmenopausal women, the symphysis donor site did not demonstrate any statistically significant variation.

**Conclusion:**

Hormone-related skeletal alterations that emerge in the postmenopausal period, particularly osteoporosis, should be recognized as a potential risk determinant in the context of surgical planning. This consideration is especially relevant for oral and maxillofacial surgeons performing procedures that utilize intraoral autogenous block graft donor sites.

## Introduction

Postmenopausal osteoporosis results from decreased estrogen levels, which increase osteoclast activity. This imbalance increases bone resorption and reduces bone formation, leading to significant bone loss, especially around menopause onset. As a result, bone mineral density and microarchitectural integrity decrease, increasing fracture risk [1]. Within the United States, osteoporosis prevalence—based on dual-energy X-ray absorptiometry (DXA) diagnostic thresholds—is estimated at roughly 20% among women aged over 50 years, increasing to approximately 30% in those aged 65 years and above [2, 3]. During the postmenopausal phase, women experience a marked acceleration in bone loss, most prominently within the initial 5–10 years, after which the decline persists over time [4].

Bone mineral density (BMD) has been evaluated using various imaging modalities, including techniques such as quantitative computed tomography (QCT), quantitative ultrasonography (QUS), and cone-beam computed tomography (CBCT) [5]. Because mandibular bone shares structural characteristics with the skeletal system, several studies have investigated its relationship with systemic osteoporosis [6]. Routine dental imaging, including standard radiographs and cone-beam computed tomography acquired during regular dental check-ups, may thus offer a viable approach for the preliminary detection of osteoporosis [7, 8].

Due to the high cost of DXA and the growing number of elderly edentulous patients requiring implants, CBCT may provide additional information about bone quality without additional imaging. Previous studies have shown that CBCT can provide useful information about trabecular bone structure and relative bone density under standardized conditions [9, 10].

Management of bone deficiencies in the oral and maxillofacial region remains a major challenge in maxillofacial surgery. The restoration of bone within the cranio-maxillofacial complex can be accomplished through the implementation of diverse surgical approaches and biomaterials.

A wide range of strategies for bone augmentation, along with various biomaterials, have been documented in the literature; selecting the most appropriate approach is guided primarily by the defect’s anatomical site and dimensions, with the ultimate goal of ensuring sufficient bone volume to support implant placement. In oral surgery, autogenous bone is considered the gold standard because of its biocompatibility and osteogenic potential [11–13].

In the context of oral and maxillofacial bone grafting, autogenous grafts can be procured from multiple anatomical donor regions. These include the anterior and lateral portions of the mandibular ramus, the buccal zone in proximity to the third molars, the lingual cortical plate of the mandible, as well as structures such as the zygomatic bone, maxillary tuberosity, hard palate, coronoid process, and the mandibular symphysis [14, 15].

This study seeks to assess, through radiological analysis, both the quality and volume of corticocancellous bone grafts retrievable from intraoral donor sites—including the maxillary tuberosity, mandibular ramus, and mandibular symphysis—in postmenopausal women, while also offering predictive data intended to support oral and maxillofacial surgeons in the preoperative planning for this specific patient population.

## Materials and methods

The study protocol was reviewed and approved by the Clinical Research Ethics Committee of the Faculty of Dentistry at Dicle University (Diyarbakır, Turkey) during the session on 25 October 2023 (approval number: 2023-36) and was carried out in full compliance with established ethical guidelines. This retrospective study was conducted using anonymized archived CBCT images and clinical records obtained during routine clinical care. According to institutional and ethics committee regulations, no additional informed consent was required or obtained for the use of these retrospective data for research purposes. However, written informed consent for CBCT imaging had been obtained at the time of routine clinical examination as part of standard diagnostic procedures.

The study retrospectively reviewed records of patients presenting to the Department of Oral and Maxillofacial Radiology at the Faculty of Dentistry, Dicle University, from March 2021 to December 2023. These patients underwent cone-beam computed tomography (CBCT) imaging for diverse clinical reasons, such as evaluation of impacted teeth, diagnosis of maxillofacial pathologies, or planning of implant treatments. The CBCT scans were obtained for routine clinical purposes and not specifically for bone density assessment.

All CBCT images were obtained using the same device (I-CAT Vision™ Imaging Science International, Hatfield, USA) and were acquired by the same radiology technician. To ensure standardization during CBCT imaging, the reference points specified by the manufacturer were strictly followed. During patient positioning, it was confirmed that the vertical reference line of the device was parallel to the patient’s sagittal plane and that the horizontal reference line passed through the Frankfurt plane and remained parallel to the ground. The acquisition parameters were set with a voxel size of 0.3 mm, 13 × 16 cm field of view, a tube current of 5 mA, a tube voltage of 120 kV, and an exposure time of 9.6 s.

The study was designed with two groups:

- Group 1: Thirty female patients between the ages of 20 and 40 who had not entered menopause, as determined by a gynecologist.
- Group 2: Thirty female patients over the age of 55, diagnosed by a gynecologist as having entered menopause, and with at least five years since the onset of irregular periods.

Patients with a history of cancer, substance use (including smoking, alcohol consumption, or other drugs), diabetes mellitus, a history of periodontitis, thyroid dysfunction, or those who had undergone chemotherapy were excluded from the study. Data related to systemic diseases, smoking and alcohol use, periodontal history, medication records, and previous oncological treatments were retrospectively retrieved from the institutional electronic medical record system and archived patient charts.

### Identification of autogenous block graft donor sites

#### Mandibular ramus

In the case of ramus-derived autogenous grafts, the anatomical limits of the donor site were established as follows: anteriorly, beginning 2 mm distal to the second molar; posteriorly, at the first section where the ascending ramus is fully visualized; inferiorly, positioned 2 mm above the course of the inferior alveolar nerve; and medially, located 2 mm lateral to the inferior alveolar nerve.

#### Maxillary tuberosity

For autogenous grafts obtained from the maxillary tuberosity, the donor region was defined according to the following anatomical boundaries: anteriorly, starting 2 mm distal to the second molar; posteriorly, extending to the junction between the maxilla and the pterygoid process; and superiorly, bounded by the floor of the maxillary sinus.

#### Mandibular symphysis

For autogenous grafts obtained from the mandibular symphysis, the donor site was anatomically delineated as follows: superiorly, positioned 5 mm below the apices of the canine teeth; inferiorly, extending to the pogonion; laterally, starting 5 mm anterior to the mental foramina; and medially, limited by the lingual cortex.

#### Dental volumetric tomography procedure

The CBCT images of all patients included in the study were evaluated by the same specialist using the I-CAT imaging software. CBCT grayscale values provided by the I-CAT software were used as an indicator of bone density and were expressed in Hounsfield Units (HU). It should be emphasized that HU values derived from CBCT are not directly comparable to those obtained from conventional medical computed tomography (CT), since CBCT gray values may be influenced by device characteristics, acquisition parameters, beam hardening, and scatter radiation. However, since all scans were obtained using the same device and imaging protocol, these values were considered suitable for relative comparisons between groups and anatomical regions. The Hounsfield unit (HU) was used as the radiomorphometric index. For volume calculation, the Cavalieri principle—one of the stereological methods—was applied. The acquired images were examined at 0.3 mm slice intervals, and the surface area of each section was measured using the planimetric method with ImageJ software (ImageJ, version 1.37v; http://rsb.info.nih.gov/ij/). The sum of these areas was then used to calculate the total volume using the following formula:

$$\mathrm{Vref}=\sum\;\mathrm{ai}\;\mathrm{x}\;\mathrm{t},$$

In the formula, Vref represents the total or reference volume of the structure of interest; a.i. denotes the total surface area of the structural projection(s) in the *i*th section; and t represents the mean slice or section thickness. The radiomorphometric index and volume measurements were obtained from sections derived from the panoramic and implant modes of the CBCT images.

### Measurement of radiomorphometric parameters

#### Evaluation of hounsfield units

Bone density was assessed using Hounsfield Units (HU) and classified as follows:

- D1: >1250 Hounsfield units,
- D2: 850–1250 Hounsfield units,
- D3: 350–850 Hounsfield units,
- D4: 150–350 Hounsfield units,
- D5: <150 Hounsfield units [13].

Based on previous literature, HU values were further categorized into five density classes (D1–D5). Within the context of CBCT imaging, this classification should be interpreted as an approximate categorization of relative bone density rather than an absolute measurement of bone mineral density. Since CBCT-derived grayscale values are affected by device-specific and acquisition-related factors, these HU ranges should primarily be considered suitable for intra-study comparative analyses performed under standardized conditions.

The interpretative framework used for CBCT-derived HU measurements in the present study is summarized in Table 1.

**Table 1:** Interpretation framework for CBCT-derived hounsfield unit measurements used in the present study

Parameter	Interpretation in the Present Study
CBCT-derived HU values	Relative radiographic density indicator
Absolute bone mineral density assessment	Not possible
Comparison between study groups	Appropriate
Comparison between anatomical donor regions	Appropriate
Generalization to other CBCT devices or protocols	Limited
Correlation with DXA/QCT measurements	Not performed
Clinical interpretation	Relative estimation of bone quality characteristics

### Statistical analysis

The normality of the data distribution was assessed using skewness–kurtosis values and the Shapiro–Wilk test. Descriptive statistics were presented as mean and standard deviation for quantitative variables, and as frequency and percentage for qualitative variables. For comparisons between two independent groups, the Student’s t-test was used for normally distributed data, whereas the Mann–Whitney U test was applied for non-normally distributed data. The paired t-test was used to compare parameters measured on the right and left sides within the age groups. The level of statistical significance was set at *p* ≤ 0.05, and all hypotheses were tested using a two-tailed approach. Statistical analyses were performed using SPSS version 23.0 (IBM Corp., Released 2015, IBM SPSS Statistics for Windows, Version 23.0, Armonk, NY: IBM Corp.).

The power analysis performed (GPower 3.1.9.7) predicted a large effect size (d = 0.75) between pre- and post-menopausal women’s CBCT parameters. With a 5% significance level and 80% power, a total of 58 participants (29 per group) were deemed sufficient. The study was completed with a total of 60 patients, considering loss and image quality factors.

To assess the repeatability of the measurements, 20% of the total sample (*n* = 12) was re-analyzed by the same observer three weeks after the initial measurement. The intra-observer reliability (ICC) value was found to be 0.94 (95% CI: 0.91–0.97), indicating that the measurements were highly repeatable.

## Results

A total of 60 participants were enrolled in the study. The mean age was 44.88 ± 13.47 years, with a range of 22 to 62 years. The influence of menopausal status on intraoral autogenous block graft donor sites was evaluated, and the findings are summarized in Table 2.

**Table 2:**
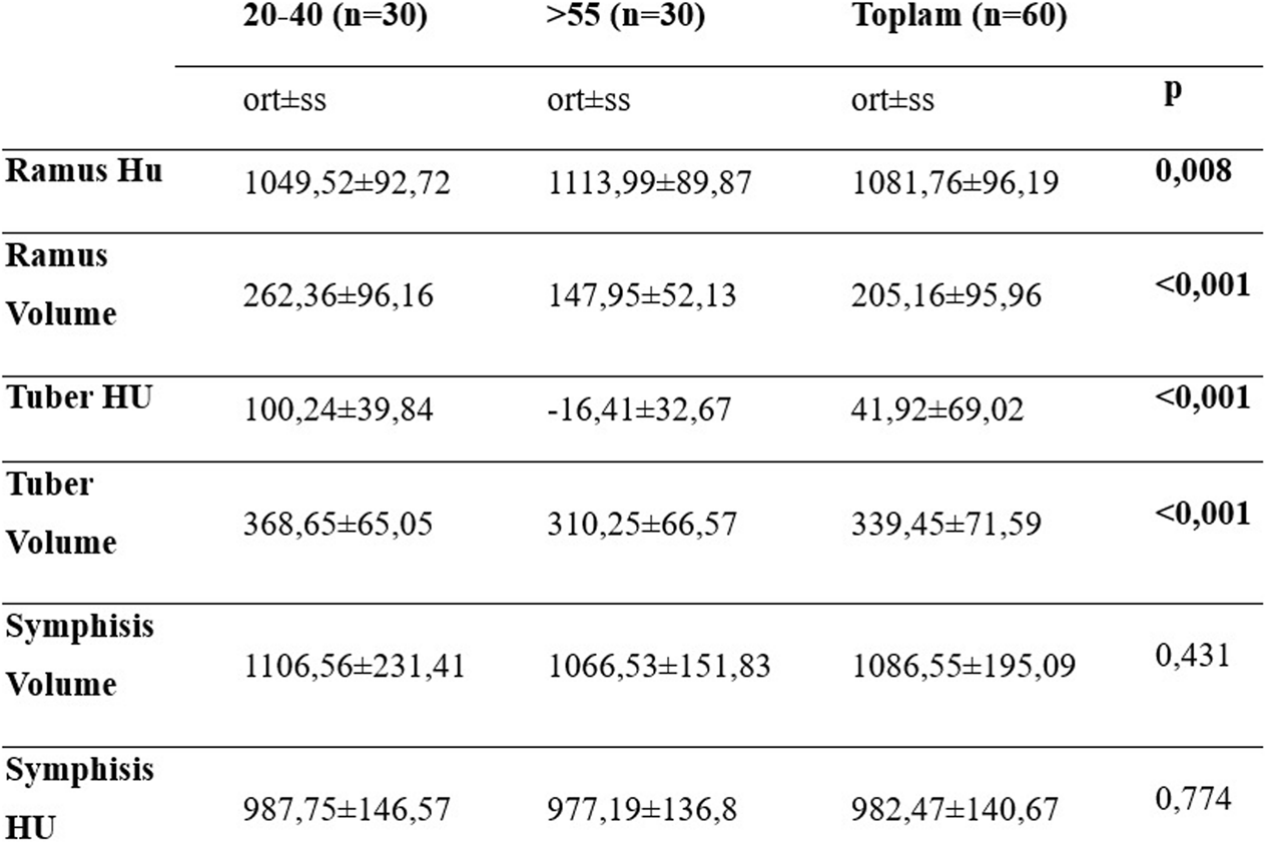
Comparison of differences in intraoral autogenous block graft donor sites between age groups

The mean Hounsfield Unit (HU) values of the ramus varied significantly across age groups. Specifically, patients over 55 years exhibited significantly higher ramus HU values than those aged 20–40 years (*p* = 0.008) (Table 2). Additionally, ramus volume differed significantly between the age groups (*p* < 0.001), with individuals aged 20–40 years showing a greater mean ramus volume compared to participants over 55 years (*p* < 0.001) (Table 2).

Both the Hounsfield Unit (HU) values and volumetric measurements of the maxillary tuberosity differed significantly across age groups (*p* < 0.001). Participants aged 20–40 years exhibited markedly higher tuberosity HU values and volumes compared to those over 55 years (*p* < 0.001) (Table 2).

In contrast, the Hounsfield Unit (HU) values and volume of the symphysis did not differ significantly between age groups, with no statistically meaningful differences detected (*p* > 0.05) (Table 2).

When the differences in parameters between the age groups were examined, the left ramus HU values were found to be similar between the groups. However, the mean right ramus HU value in patients aged over 55 years (1122.19 ± 136.79) was significantly higher than that observed in patients aged 20–40 years (1023.01 ± 107.17).

Furthermore, the mean left and right ramus volumes were significantly higher in patients aged 20–40 years compared to those aged over 55 years (*p* < 0.001) (Table 3).

**Table 3:**
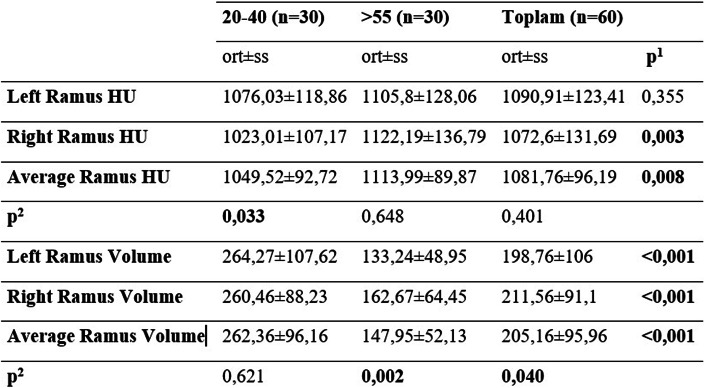
Comparison of the effects of menopause on right and left ramus volume and HU values

In patients aged 20–40 years, the mean Hounsfield Unit (HU) values of both the left and right maxillary tuberosities were significantly higher than those observed in individuals over 55 years. Furthermore, the mean left tuberosity volume in the 20–40-year age group (373.65 ± 66.26) was significantly greater than that of patients over 55 years (300.86 ± 84.16) (Table 4).

**Table 4:**
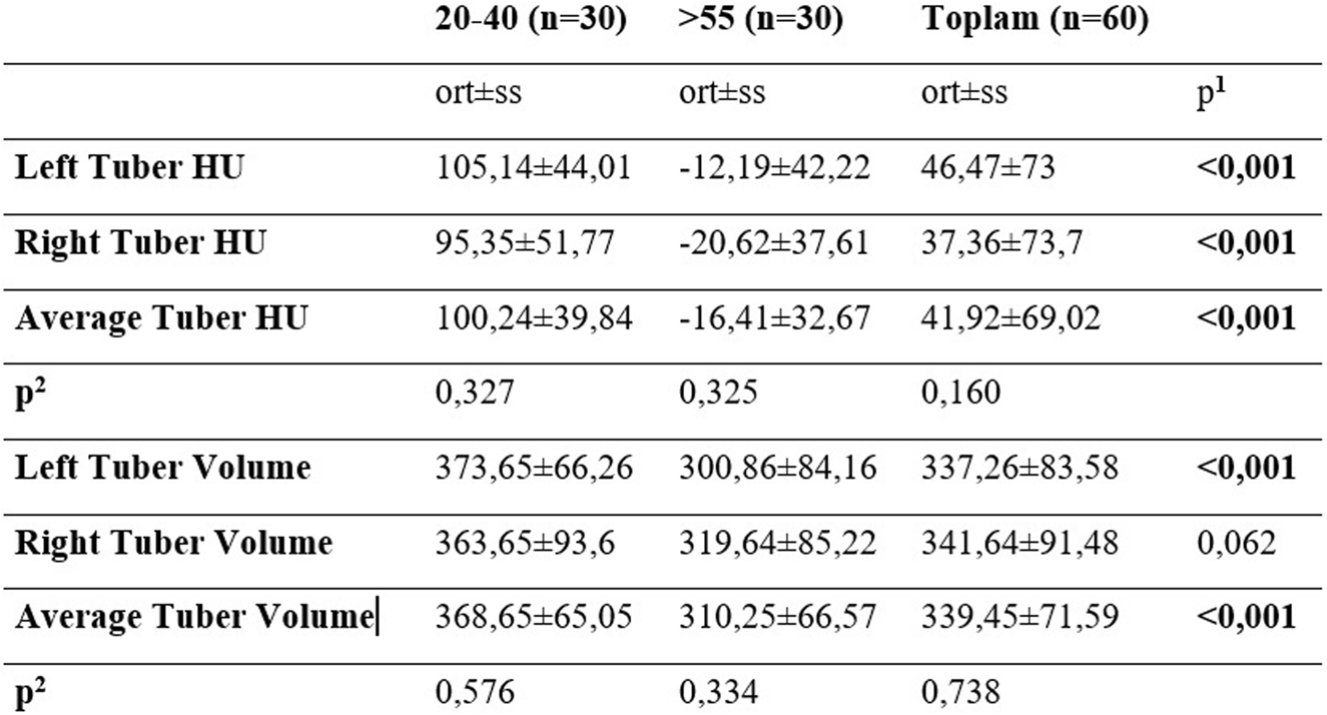
Examination of the effects of menopause on left and right tuberosity volume and HU values

## Discussion

Globally, as nations have advanced in terms of development, the average lifespan of human populations has shown a consistent upward trend. This increase in longevity has been accompanied by a higher incidence of chronic health conditions. Furthermore, osteoporosis has emerged as a major public health issue, especially among postmenopausal women, due to the heightened susceptibility to bone fractures.

Shaw et al. [16] examined how aging impacts mandibular morphology and facial esthetics. Their findings indicated that, in both male and female participants, bigonial width and ramus width remained largely unchanged with age. In contrast, ramus height exhibited a significant decline as age increased across both sexes. The study also assessed mandibular body height and length, both of which showed notable reductions over time, with the decrease being particularly pronounced in elderly women. Consistent with these observations, our study similarly identified a volumetric decrease in the mandible associated with advancing age [16].

In their 2013 study, Mikami et al. investigated the microarchitecture and turnover of alveolar bone in both premenopausal and postmenopausal women. The results indicated that women in the late postmenopausal stage had significantly lower alveolar bone volume and bone mineral density compared to premenopausal women. In contrast, no significant differences were observed between early postmenopausal and premenopausal women [17].

Takaishi et al. examined the relationships among periodontitis, systemic skeletal alterations, and mandibular bone loss in postmenopausal Japanese women. Their study analyzed CBCT scans of 40 postmenopausal participants, aged 50 to 69 years, presenting with minimal to mild periodontal disease. The findings showed that postmenopausal women experienced decreases in alveolar bone volume and bone mineral density, with the magnitude of these reductions increasing progressively with age [18].

Tanaka et al. investigated both radiographic and histological alterations in the mandibular cortices of ovariectomized monkeys. The study comprised twelve adult female cynomolgus monkeys, with radiographic assessments conducted 76 weeks following ovariectomy. The results demonstrated that the observed mandibular cortical bone loss, accompanied by elevated bone turnover, was primarily attributable to estrogen deficiency [19].

Ko et al. carried out a prospective study to evaluate and compare crestal cortical bone thickness at dental implant sites in menopausal versus non-menopausal women. Their findings revealed that older women exhibited reduced crestal cortical bone thickness, with the most pronounced thinning observed in the posterior maxilla, relative to younger participants. Additionally, across both age groups, the posterior region of the mandible consistently exhibited the highest crestal cortical bone thickness, while the posterior maxilla displayed the lowest values [20].

In their 2018 study, Balto et al. investigated the association between BMD, panoramic radiomorphometric indices, and the identification of osteoporosis in postmenopausal women. The research cohort consisted of 431 Saudi women with a mean age of 57.8 years. The results demonstrated that the age at menopause onset did not differ significantly among participants; however, the likelihood of osteoporosis increased with the duration of the postmenopausal period [21].

Zhang et al. observed that autogenous grafts harvested from the mandibular ramus of male patients exhibited greater height and width compared to those obtained from female patients. Additionally, their study indicated that patient age did not significantly influence either the height or width of the grafts [22].

Li et al. [23] investigated age-related changes in condylar bone mineral density and trabecular structure in women using cone-beam computed tomography (CBCT), focusing particularly on postmenopausal alterations. CBCT scans were collected from 160 female participants, who were divided into four age-based groups: 20–29 years, 30–39 years, premenopausal, and postmenopausal. The study found that condylar bone volume relative to tissue volume, trabecular number, and trabecular thickness all declined progressively with age, with the postmenopausal group showing significantly lower values compared to the younger and premenopausal groups [23].

Unlike conventional medical CT, CBCT systems do not provide fully standardized HU measurements because grayscale values may be influenced by factors such as device type, exposure parameters, beam hardening, scatter radiation, and image reconstruction algorithms [24]. Therefore, the HU values obtained in the present study should not be interpreted as absolute bone mineral density measurements. Nevertheless, because all CBCT scans were acquired using the same device and standardized acquisition parameters, comparisons between anatomical regions and between study groups remain valid within the methodological framework of this study.

Furthermore, the present study did not calibrate CBCT values using a phantom reference system, nor were the findings correlated with systemic bone mineral density measurements such as dual-energy X-ray absorptiometry (DXA) or quantitative computed tomography (QCT). Consequently, the HU values reported herein should be regarded as relative indices rather than direct representations of absolute bone mineral density. Future studies combining CBCT-based intraoral donor site assessment with DXA or QCT measurements may help clarify the extent to which CBCT-derived measurements reflect systemic skeletal status.

Unexpectedly, postmenopausal women showed higher ramus HU-equivalent values despite lower ramus volume. Initially, a reduction in bone density in postmenopausal individuals would be expected due to estrogen deficiency and age-related skeletal deterioration. However, the present findings suggest a more complex structural adaptation in the mandibular ramus.

One possible explanation may be the relative corticalization of the ramus region with advancing age. Progressive trabecular bone loss accompanied by preservation or relative predominance of cortical bone may lead to increased grayscale values within a smaller total bone volume. In addition, the ramus region is continuously exposed to mechanical loading associated with masticatory muscle activity, which may contribute to the preservation of cortical density in accordance with Wolff’s Law.

However, imaging artifacts, beam hardening, patient positioning, and device-related factors may also have influenced these findings. Therefore, the unexpectedly higher HU values observed in the postmenopausal ramus region should be interpreted cautiously and validated by future studies using standardized quantitative imaging methods.

Based on the present findings, the mandibular ramus may demonstrate a relative preservation of radiographic density despite age-related volumetric reduction. This observation may partially be explained by continuous mechanical loading generated by masticatory muscle activity, consistent with Wolff’s Law [25, 26]. However, unlike an actual preservation of bone quantity, the current results demonstrated reduced ramus volume accompanied by unexpectedly higher HU values in postmenopausal women. Therefore, these findings should be interpreted as a potential alteration in bone composition or corticalization rather than true structural stability.

## Conclusion

The present study demonstrated that menopause-related physiological alterations adversely affect the quantity and radiographic density characteristics of certain intraoral autogenous block graft donor sites, particularly the mandibular ramus and maxillary tuberosity. In contrast, the symphysis region appeared relatively unaffected.

Although CBCT-derived HU measurements have methodological limitations and should not be interpreted as absolute bone mineral density values, standardized intra-study comparisons revealed significant differences between premenopausal and postmenopausal women. These findings suggest that menopausal status may influence both graft volume and relative bone density characteristics in intraoral donor regions.

Therefore, menopause and associated skeletal alterations should be considered during surgical planning for reconstructive procedures involving autogenous bone grafts in postmenopausal patients. Further studies integrating CBCT findings with systemic bone density assessment methods such as DXA or QCT are warranted.

## Data Availability

Research results supporting the findings of this study are available from Dicle University Faculty of Dentistry; however, there are restrictions on the availability of this data, as the data were used under license for the present study and are therefore not publicly available. Nevertheless, the data may be obtained upon request and with the permission of Dicle University Faculty of Dentistry.

## Abbreviations

CBCT: Cone-beam computed tomography
HU: Hounsfield unit
DXA: Dual-energy X-ray absorptiometry
QCT: Quantitative computed tomography
BMD: Bone mineral density
